# Exploring the role of place attachment in shaping sustainable behaviors toward marine cultural heritage: a case study of Dongmen village in Fujian Province, China

**DOI:** 10.3389/fpsyg.2025.1476308

**Published:** 2025-01-29

**Authors:** Wenchao Cai, Zhangwen Shu, Yisi Liu

**Affiliations:** ^1^Laboratory of Archaeometry, Xiamen University, Xiamen, China; ^2^School of History and Cultural Heritage, Xiamen University, Xiamen, China; ^3^School of Physical Education, Yunnan Normal University, Kunming, China; ^4^Xi'an Institute of Cultural Relics Protection and Archaeology, Xi'an, China

**Keywords:** place attachment, sustainable behavior, faith groups, cultural heritage construction, emotions

## Abstract

Local residents’ sustainable behaviors associated with cultural heritage are of paramount importance, however the complexities involved are yet to be disentangled. In this study, the concept of the sustainability of residents’ cultural heritage behaviors based on the theory of place attachment is investigated, with a particular focus on religion-related components, using the case of Dongmen village in Fujian Province, China. An attempt is made to answer how residents’ place attachment affects the sustainability behavior of cultural heritage construction and homebuilding. Based on the theory of place attachment, the sustainability of cultural heritage behavior from the perspective of local residents is employed. It is evident that the residents of Dongmen village make use of local knowledge and indigenous resources, and they proactively use associations of the elderly, faith groups, family networks, and overseas relationships. The results of this research show that the sustainable behaviors of Dongmen village residents toward cultural heritage construction are influenced by their feelings of place attachment, which subsequently influence homeland construction actions that, in turn, shape person–place relationships.

## 1 Introduction

Place attachment is a significant concept in environmental psychology and socio-cultural studies (Milligan, 1998; Hidalgo and Hernandez, 2001; Hernández et al., 2007; Scannell and Gifford, 2010; Lewicka, 2011). It is commonly defined as a profound emotional bond between individuals and specific places(Giuliani and Feldman, 1993; Hay, 1998). As a complex psychological and social phenomenon(Mazumdar, 2005), place attachment not only manifests as a sense of belonging and dependence on a particular space but also involves a deep identification with the symbolic meaning of the place(Walker and Chapman, 2003; Morgan, 2010; Massey, 1994).

Place attachment is a core concept within place theory, with its origins rooted in the work of earlier phenomenological scholars. Eliade (1959) and Bachelard (1964) emphasized the emotional connections and bonds people have with spaces, particularly focusing on sacred spaces and homes. In the 1970s, research on crowding gained momentum, spurring interest in understanding the meaning of place attachment (Altman and Low, 1992). During this period, social scientists began to underscore the importance of human variables in addressing environmental issues. Early social psychological studies on environmental matters frequently emphasized people’s attitudes, and after the OPEC oil embargo of the 1970s, the focus shifted toward examining behaviors relevant to the sustainable use of natural resources (Becker et al., 1981; Gonzales et al., 1988; Kurz, 2002).

In the recent decades, place attachment has gained considerable scientific attention (Scannell and Gifford, 2010). This interest began in humanistic geography and architectural phenomenology in the 1970s, evolving over the past two decades, particularly influenced by the phenomenologies of place advanced by environmental thinkers such as Casey (2009), Malpas (2001), Mugerauer (1994), and Stefanovic (1998). Recent research has offered a provocative new understanding of human life and experience, shedding light on how attachment to place can influence behavior (Seamon, 2018). The effect of place attachment on pro-environmental behavior has been explored by researchers in both social and environmental psychology (Ramkissoon et al., 2012), and it is often cited as a prerequisite for fostering pro-environmental behaviors (Lee, 2011).

As the study of place attachment evolved, the focus expanded to incorporate more nuanced understandings of the emotional connections between people and places. Tuan (1974) introduced the term “topophilia,” describing a broad spectrum of affective ties with the physical environment. Following this, Stokols and Shumaker (1981) extended the concept to include expectations of stability, greater knowledge of the locale, and behaviors aimed at enhancing or maintaining the place. Altman and Low (1992) suggested that place attachment is multifaceted, contributing to individual, group, and cultural self-definition. Research by Williams and Vaske (2003) and Lewicka (2011) has specifically explored the emotional bonds that individuals develop with particular places, highlighting the positive connection that place attachment fosters. Despite these advancements, scholars continue to call for more research to better understand the mechanisms underlying place attachment, particularly in relation to residents’ attitudes (Eusébio et al., 2018; Ramkissoon, 2020; Strandberg et al., 2020).

Place attachment plays a significant role as an indicator in achieving sustainable development goals (Hemani et al., 2017; Larimian et al., 2020; Anestad et al., 2024). Defined as the emotional bond between individuals or groups and specific places, place attachment enhances community members’ sense of responsibility for the sustainable protection and development of their locality (Mulaphong, 2022; Bernardo et al., 2023; Han et al., 2024; Darko and Halseth, 2025). It also motivates long-term positive actions to preserve ecological, cultural, and social resources (Baah et al., 2024; Lin et al., 2024). Specifically, Bernardo et al. (2023) argued that a strong sense of place identity fosters a sense of ownership and responsibility toward the community and environment, contributing to transformative change within the framework of sustainable development. Darko and Halseth (2025) highlighted the interaction between place attachment and social capital as a critical factor influencing community responses to economic transitions. Furthermore, place attachment strengthens emotional value recognition of places, providing enduring social motivation and cultural support for sustainability (Wei et al., 2022).

Place attachment significantly influences residents’ involvement in managing heritage resources and tourism development. Strzelecka et al. (2017) found that residents with strong place attachment are more likely to engage in these activities, and the recreational and aesthetic value of places further strengthens this bond (Jayakody et al., 2024). However, there is limited understanding of how locals develop attachments to their communities, especially when tourism impacts culturally important sites (Hoang et al., 2020). In the context of cultural heritage, place attachment shapes how individuals engage in resource management and political actions, as their connection to a place often defines their identity (Lee, 2011). Given that heritage tourism holds different meanings for different people, understanding place attachment in a local context is crucial (Vong, 2015).While residents play a key role in sustainable heritage management, little is known about how place attachment develops in tourism settings. Understanding residents’ attachment to heritage places is essential to clarifying their attitudes and behaviors toward tourism (Hoang et al., 2020). Although residents’ sustainable behaviors are critical, further research is needed to explore how experiences at heritage sites shape both place attachment and sustainable behaviors (Ballantyne et al., 2011).

Although previous studies have explored the relationship between sustainable behaviors and the environment(Kurz, 2002; Zelenski et al., 2015; Chwialkowska et al., 2020; Davari et al., 2024), there has been insufficient understanding of how residents’ emotional connections drive sustainable behaviors(Jacobs and McConnell, 2022). While place attachment theory has been explored in various cultural contexts (Hall, 2018; Hoang et al., 2020; Wang, 2023), research on sustainable behaviors within the specific context of Chinese culture remains limited (Gursoy et al., 2019). By focusing on the rural areas of southeastern coastal China, this study highlights the unique characteristics of place attachment in the context of maritime civilization. These areas have strong historical and cultural ties and demonstrate significant cross-regional influences (Yi et al., 2023), emphasizing the importance of emotional connections in shaping sustainable practices (Dickinson et al., 2016).

This study aims to deepen our understanding of how residents’ place attachment influences their sustainable behaviors related to cultural heritage and homebuilding. By integrating the theory of place attachment with principles of sustainable development, this research seeks to explore the emotional connections that drive residents’ participation in preservation and reconstruction activities. To achieve this, the study proposes three specific research objectives: (1) investigate the nature and dimensions of residents’ emotional bonds with their cultural heritage, identifying key factors that foster strong place attachment and shape their attitudes toward heritage preservation and homebuilding; (2) analyze how these feelings of place attachment translate into concrete actions for preserving and reconstructing cultural heritage, highlighting both individual and community-level behaviors; and (3) Provide strategic recommendations for heritage planners by highlighting how residents’ place attachment, coupled with their use of local knowledge, social networks, and indigenous resources, can contribute to heritage conservation and reconstruction efforts, thereby promoting sustainable development in culturally significant areas.

## 2 Materials and methods

### 2.1 Study area

**Figure 1:**
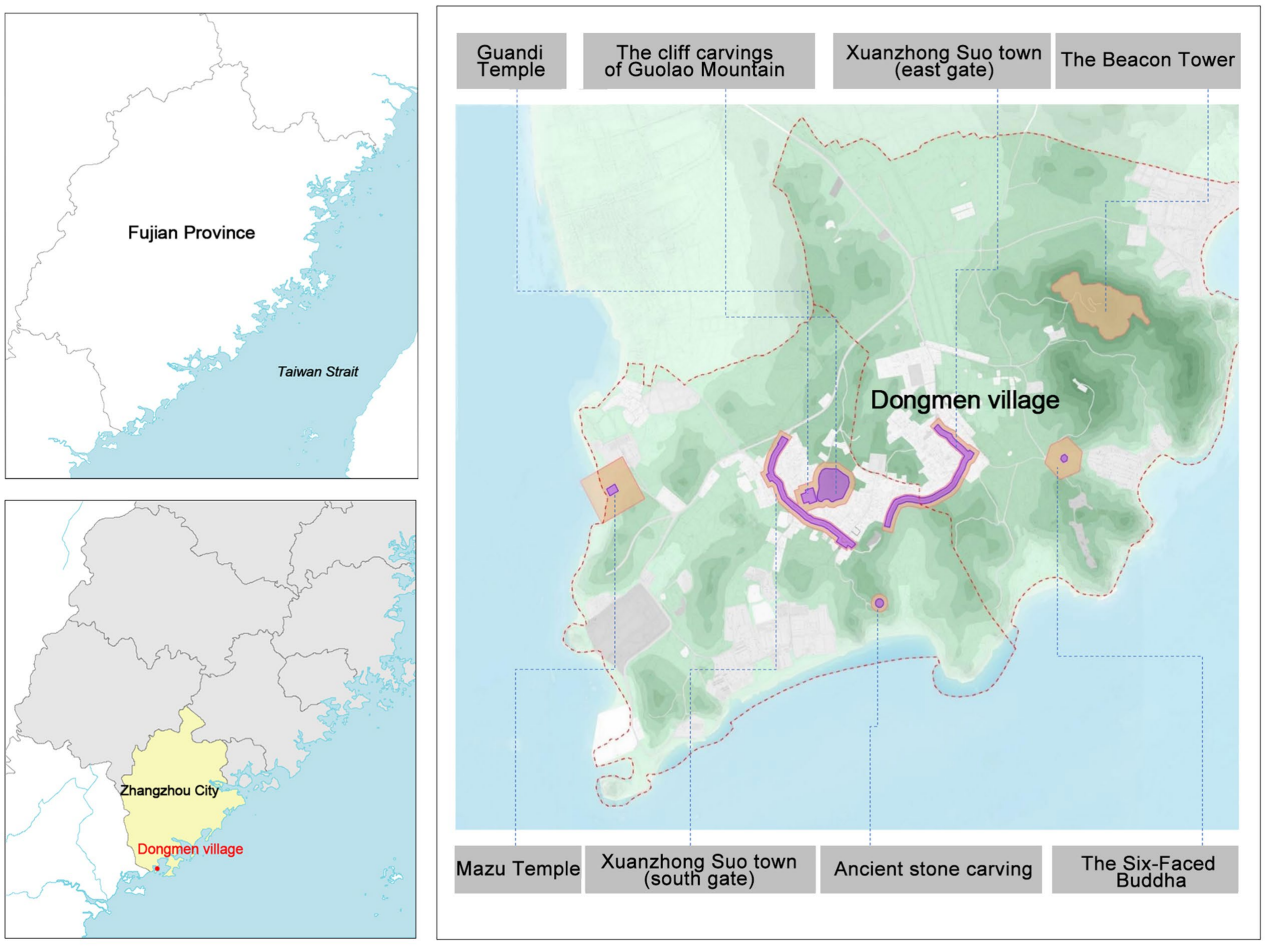
Research area and distribution of local heritage in Dongmen village (redrawn from the 1:4,500,000 Fujian Province map prepared by the Fujian Provincial institute of cartography and the Dongmen village planning).

**Figure 2:**
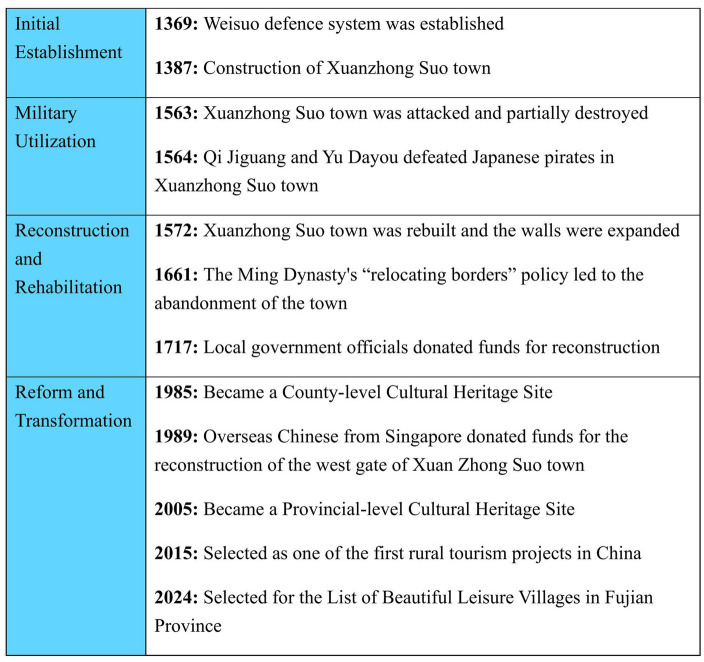
Historical overview of Dongmen village.

Dongmen village, formerly known as Xuanzhong Suo Town, is situated in the southern part of Meiling Peninsula, Zhao’an County, Zhangzhou City, Fujian Province, China. It occupies a strategic position at the confluence of the South China Sea and the East China Sea, characterized by its rugged terrain (Figure 1). Xuanzhong Suo town was built in 1387, embedded in the social texture of its role in the coastal defense forts system of the Ming dynasty, which impacted its regional politics and economy. During the Ming dynasty, coastal defense constituted a critical issue within the maritime policies of its rulers (Lee, 2017). The coastal regions of China began to be raided by particular groups of Japanese, and other pirates known as Wokou. The Hongwu Emperor ordered the establishment of a chain of garrisons all along the coast (Torck, 2015). In 1369, a Ming expedition was sent by the founding emperor to capture Guangdong from a contesting force. After the pacification of the region, the Weisuo defense system was established along the coast (Ng, 2017). According to the Zhao’an County Chronicle, Xuanzhong Suo town, located in Zhao’an County, is on a mountainous terrain with a circumference of 1800 meters, a wall surface of 3 to 3.5 meters, and a height of 6 meters. There were four gates on the east, west, north, south, and west. The east and west gates blocked the sea, the north gate was the access road, and the south gate was the plug (Chen and Wu, 2000). Xuanzhong Suo town was sunk by Japanese pirates in 1563 and rebuilt in 1572. Due to the Japanese invasion, in the ninth year of the Wanli period (middle of the Ming dynasty, 1581), the TongShan Zhejiang military camp was set up as defense against areas from TongShan to HangZhong, Zhao’an, and other coastal areas. The famous anti-Japanese generals Qi Jiguang and Yu Dayou defeated Japanese pirates in Xuanzhong Suo town and the surrounding waters (Figure 2).

Dongmen village is a cultural heritage in the making. It is an important starting point for the Maritime Silk Road. Since the Song dynasty, it has been the main port of foreign trade in Zhangzhou, Fujian Province. There are cultural relics in Dongmen village, including the Mazu Temple, Guandi Temple, cliff carvings of Guolao Mountain, and the Ancestral Hall, among other notable examples (Figure 1). The website of the People’s Government of Fujian Province shows that in 2005 the circumvallation of Xuanzhong Suo town was listed by the Government as the sixth batch of provincial cultural relics protection units, which expresses the critical position of Xuanzhong Suo town in the coastal defense forts system. Today, many families are living and working in Dongmen village, and some of them are descendants of the officials and soldiers who guarded the coast. Residents’ livelihoods are mainly based on fishing, agriculture, and tourism.

### 2.2 Research methods

#### 2.2.1 Phases of ethnographic fieldwork and data gathering

This study employed ethnographic fieldwork methods, including in-depth interviews and participant observation, to explore the relationship between place attachment and sustainable behaviors in the context of cultural heritage. As pioneered by Malinowski, ethnographic fieldwork provides profound insights into the role of emotions in social life and the dynamics of cultural phenomena (Malinowski, 1992; Beatty, 2014). It seeks to outline the social constitution and identify the patterns and regularities of cultural practices while separating them from irrelevant details.For this study, local literature and materials were collected through participatory observation and in-depth interviews, with a focus on the ecological, political, and cultural environments of the research site. Guided by the holistic perspective of cultural anthropology, the research emphasized the interconnections and interdependence of various aspects of human experience, encompassing both biological and cultural dimensions, past and present (Haviland, 2010).

In the first phase of the research, our research team visited Dongmen village in June 2018 to gain an initial understanding of the village’s geographical location, ecological environment, and settlement characteristics. We focused on the residents’ livelihood patterns and the current status of cultural heritage preservation. Through village visits, we documented the layout of houses, the road network, and the distribution of public facilities, as well as gained insights into the villagers’ lifestyles and community structure. Informal conversations with villagers facilitated initial connections with several residents involved in lodging and commerce. Additionally, we established contact with local heritage conservation departments through direct visits.

The second phase, conducted in June 2019, concentrated on the sustainability of residents’ cultural practices. This phase involved in-depth interviews with local residents, with initial participants selected randomly based on their willingness to participate during the survey period. These participants were subsequently asked to recommend potential interviewees. Furthermore, a government official accompanying the team recommended three local residents, noting their lifelong status as inhabitants who have witnessed the interaction between the community and the evolving heritage landscape over more than 50 years. An important factor in their selection was their fluency in Mandarin, which facilitated direct communication with the researchers. During this phase, we also visited the local government archives to review historical documents and policy papers related to Dongmen village and engaged with staff from the village committee and community service center to understand the village’s administrative management and social services. The researchers in this study also rented a room from one of the participants to facilitate closer communication with them. During this period (the second phase), the researchers engaged in aspects of the cultural heritage development process in Dongmen village, which included attending meetings on tourism infrastructure decision-making, discussions on cultural heritage conservation planning, and programs for artifact restoration. When it is necessary to record the process of cultural heritage protection (such as rebuilding the ancestral temple), video recording was conducted with the consent of all parties to collect qualitative data. In-depth interviews provided valid data on how residents’ place attachments influence sustainable behavior toward cultural heritage.

#### 2.2.2 Semi-structured interviews and ethical considerations

**Figure 3:**
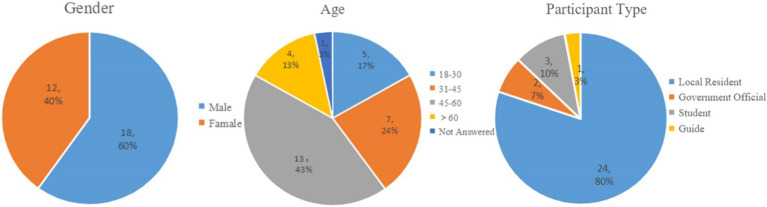
Key characteristics of the interview sample (*N* = 30).

We employed semi-structured interviews for both on-site and off-site groups, conducting a total of 30 in-depth interviews to ensure comprehensive coverage of key research themes while maintaining a degree of openness in the discussions. This approach enabled us to explore individual experiences, perspectives, and social dynamics within the heritage community in depth, resulting in the collection of rich and detailed data. Figure 3 summarizes the demographic characteristics of the 30 participants, whose ages range from 19 to 67. Given that the survey was conducted mid-year, a period when many younger individuals were working away from the village, participants aged 30 and above comprised 80% of the total sample. The majority of the participants were long-term local residents.

Participants were selected based on prior knowledge and snowball sampling techniques to ensure that these interviews were representative and comprehensive, including members of residents, local government officials, and a guide (Figure 3). With consent obtained, our interviews typically lasted between 30 min to 1 h. Additionally, follow-up interviews were conducted with some participants via voice calls. Except for two government officials and the three participants they recommended, the remaining 25 participants were randomly selected at the survey site and through recommendations from other participants. The selection of key participants also takes into account their level of association with cultural heritage and their willingness to participate (Palinkas et al., 2015).The interview guide for local residents covered six themes: personal background and relationship with the village, place attachment and emotional connection, folk beliefs and cultural practices, historical memory and village transformation, cultural heritage and sustainable behavior, and the interaction between place attachment, folk beliefs, and village development (Table 1).

**Table 1:** Key interview questions for participants.

Personal background and relationship with the village	How long have you lived in this village? How many generations has your family been here?
	What does this village mean to your personal identity and family history?
	How do you participate in community activities or cultural traditions in the village? What importance do these activities have in your daily life?
Place attachment and emotional connection	What is the strongest emotion you feel towards the village? Through what experiences or events was this connection formed?
	Has the significance of this place for you changed at different stages of your life? If so, how would you describe these changes?
	How do the natural and cultural landscapes of the village influence your emotional connection? Are there specific sites or places that are particularly meaningful to you?
Folk beliefs and cultural practices	What role do folk beliefs play in the community? Are there any significant rituals or traditions that connect you to the village?
	Are there specific places in the village that hold religious or spiritual significance? How do these places contribute to the village’s identity and your connection to it?
	How have these beliefs influenced your sense of belonging or attachment to the village?
Historical memory and village transformation	Can you recall significant changes or events in the village’s past? What impact did these events have on the village?
	Which aspects of the village’s history do you think are most important? How do villagers view these historical heritages?
	As the village gradually developed into a cultural heritage site, did your memory of the village’s history change? If so, in what ways?
Cultural heritage and sustainable behavior	How do you think the villagers participate in the preservation and protection of the village’s cultural heritage? Have you personally been involved in this?
	How do you think attitudes toward the protection of the village’s history and traditions have changed before and after the village became a heritage site?
	In your opinion, how do residents’ place attachment and beliefs influence the sustainable development of cultural heritage? How are these behaviors passed on or shared among villagers?
Place attachment, folk beliefs, and village development	Since the village became a heritage site, what changes have occurred in village life? How have these changes impacted your daily life?
	Do you feel that modernization or external influences have weakened villagers’ place attachment or traditional beliefs? How have residents responded to these external changes?
	How has the development of tourism in the village affected your personal life? What is your view on the influence of tourists on village culture and its spiritual practices?

Most of the interviews were conducted entirely in Mandarin, with nine participants using some Min Nan dialect during the interviews. Real-time translation into Mandarin was provided by one of the researchers who understood both languages. They were audio recorded and transcribed. In addition to the formal interviews, the study draws on qualitative data obtained from some informal discussions with residents of Dongmen village, many of whom were at work or community events at the time of data collection. In addition, historical documents about Xuanzhong Suo town and Dongmen village were consulted, as well as folk documents such as genealogy and letters.

This study adhered to strict ethical standards to protect participants’ rights, privacy, and anonymity (Ravindran, 2019). Before conducting interviews, the research team built trust through informal interactions, enabling participants to understand the study’s purpose. Detailed information on objectives, procedures, and rights—including the right to withdraw—was provided, and informed consent was obtained verbally or in writing. To safeguard identities, pseudonyms were used, and identifying details anonymized, particularly given the diverse participant pool, including government officials and residents with varying views on cultural policies.

In the course of fieldwork, during cultural heritage documentation such as video recordings, explicit consent was secured, and alternative methods like written notes were offered to those uncomfortable with recording. The researchers resided in the village during fieldwork, fostering immersion and meaningful engagement while maintaining ethical boundaries. This approach enhanced understanding of community dynamics and strengthened participant trust.

#### 2.2.3 Summative content analysis

This study employs a summative content analysis approach to examine the interview data, field observations, and historical documents. Summative content analysis focuses on identifying key themes and patterns from qualitative data through summarization rather than deep coding, offering a holistic understanding of the research context (Hsieh and Shannon, 2005). This method allowed us to explore the influence of place attachment on residents’ sustainable behaviors within the unique cultural and geographical context of Dongmen village.

In this study, the interview data were transcribed verbatim and systematically reviewed. Through multiple readings of the interview content, we concentrated on identifying the participants’ perspectives, experiences, and emotions. The aim was to uncover meaningful insights that highlight the significance of cultural heritage to the community and its role in the preservation process. The analysis involved several key steps:

1. Theme Identification: we synthesized the data to identify key themes such as cultural heritage identity, challenges in preservation, and community participation, which provided the foundation for understanding place attachment;
2. Contextual Analysis: we analyzed how personal experiences, and the community environment influenced the participants’ views on heritage preservation (Unger, 1995);
3. Comparison and Contrast: we compared the perspectives of different participants, highlighting similarities and differences in their views on cultural heritage, which helped us understand varying community perceptions;
4. Rich Narratives: we preserved participants’ original expressions to capture their authentic voices and emotions (Brydges and Hracs, 2018), such as one participant’s remark about the discrepancies between the county’s historical records and current tourism plans. These steps allowed us to comprehensively analyze the data and gain insights into the residents’ relationships with their heritage and their sustainable behaviors toward its preservation.

## 3 Findings

### 3.1 Cultural heritage reconstruction and its role in shaping place attachment in Dongmen village

Xuanzhong Suo Town was abandoned following the implementation of the “Relocation of Boundaries” policy in the 18th year of the Shunzhi period (1661). Since then, the town has had no role in the main structure of the defense system. It has been slowly transformed into a general residential settlement space, and today, the scale and level of development remain at the level of a village. However, the spatial pattern, building types, and place names of the original Xuanzhong Suo town convey the characteristics of marine cultural heritage. These are important vehicles that enable a place to have its characteristics. They carry the folk narration and historical memory of Xuanzhong Suo town, thus, entering the discourse on cultural heritage related to the Maritime Silk Road network. Heritage reflects not just habit but a conscious choice (Lowenthal, 1998). The historical relics and marine cultural heritage associated with Xuanzhong Suo town have been reconstructed, thus, Dongmen village has attempted to become one of the core areas of the Maritime Silk Road.

As previously mentioned, Xuanzhong Suo town was built in 1387, embedded in the social texture of Zhao’an Bay, at that time, had the role of the coastal defense forts system of the Ming dynasty, which impacted the region, politics, and the economy. Nowadays, the historical relics and cultural contents related to Xuanzhong Suo town are regarded as important cultural resources. According to the Chinese studies scholar Wang Huihui, in 2006, after China’s National Cultural Heritage Administration included Quanzhou and Ningbo in the preparatory list of the Maritime Silk Road inscription, the relevant cities also addressed the issues by taking action, and Fujian Province organized relevant departments to start the declaration of Zhangzhou’s application for heritage (Wang, 2013).

Dongmen village is trying to enter the system of cultural heritage discourse related to the Maritime Silk Road and has experienced ‘deconstruction–reconstruction’ in the process of recombining cultural resources. The homeland images that the Dongmen village residents have, are influenced by the cultural heritage trend in recent years, which has been discovered by the outside world as ‘Suo towns of the Ming dynasty’ and the Maritime Silk Road. Conserving the marine cultural heritage involves the reconstruction of a series of historical landscapes related to Xuanzhong Suo town of the Ming dynasty.

*What you now call 'cultural heritage' was just our familiar living environment growing up. As children, we used to climb and play among those broken walls and ruins. In other words, before they became 'heritage,' to us older folks, they were simply part of our hometown, a source of joy, youthful joy. But as these walls and ruins became 'heritage,' it was through diverse forms of promotion that we came to understand that they also represent history. They are different from ancestral halls, yet still connected to our ancestors in some way* (Wu Fei, Male, 67 years old).

*The Maritime Silk Road—this name is also very new to me. I understand that it's part of the government's efforts to rediscover history. Where does culture come from? Of course, it comes from these historical relics left by our ancestors, which is why they are being reevaluated or restored one by one. Naturally, we welcome this, because if culture can be rediscovered, it can attract people to come and visit. Your research here is also a form of protecting these things* (Huang Yan, Male, 35 years old).

From these two interviews, it is evident that the government-led heritage promotion strategies have significantly influenced the local residents’ perceptions. This reflects the ‘dual identity’ that emerges in the process of heritage re-evaluation—heritage not only retains its function as a carrier of personal emotions but also increasingly becomes an important symbol of historical and cultural representation. Moreover, the interviews reveal a critical issue: place attachment is strongly influenced by external constructs. Since China’s accession to the World Heritage Organization, various levels of local governments and cultural heritage departments have intensified funding and cultural relic surveys, driving a series of heritage preservation movements. In 2023, the national expenditure on culture and tourism amounted to 128.04 billion yuan, an increase of 7.86 billion yuan compared to the previous year. In recent years, these preservation efforts have often been intertwined with local cultural identity (a process officially framed as “cultural confidence”), a concept proposed by the Chinese government that emphasizes the recognition, affirmation, and pride in the value of China’s own cultural heritage.

**Figure 4:**
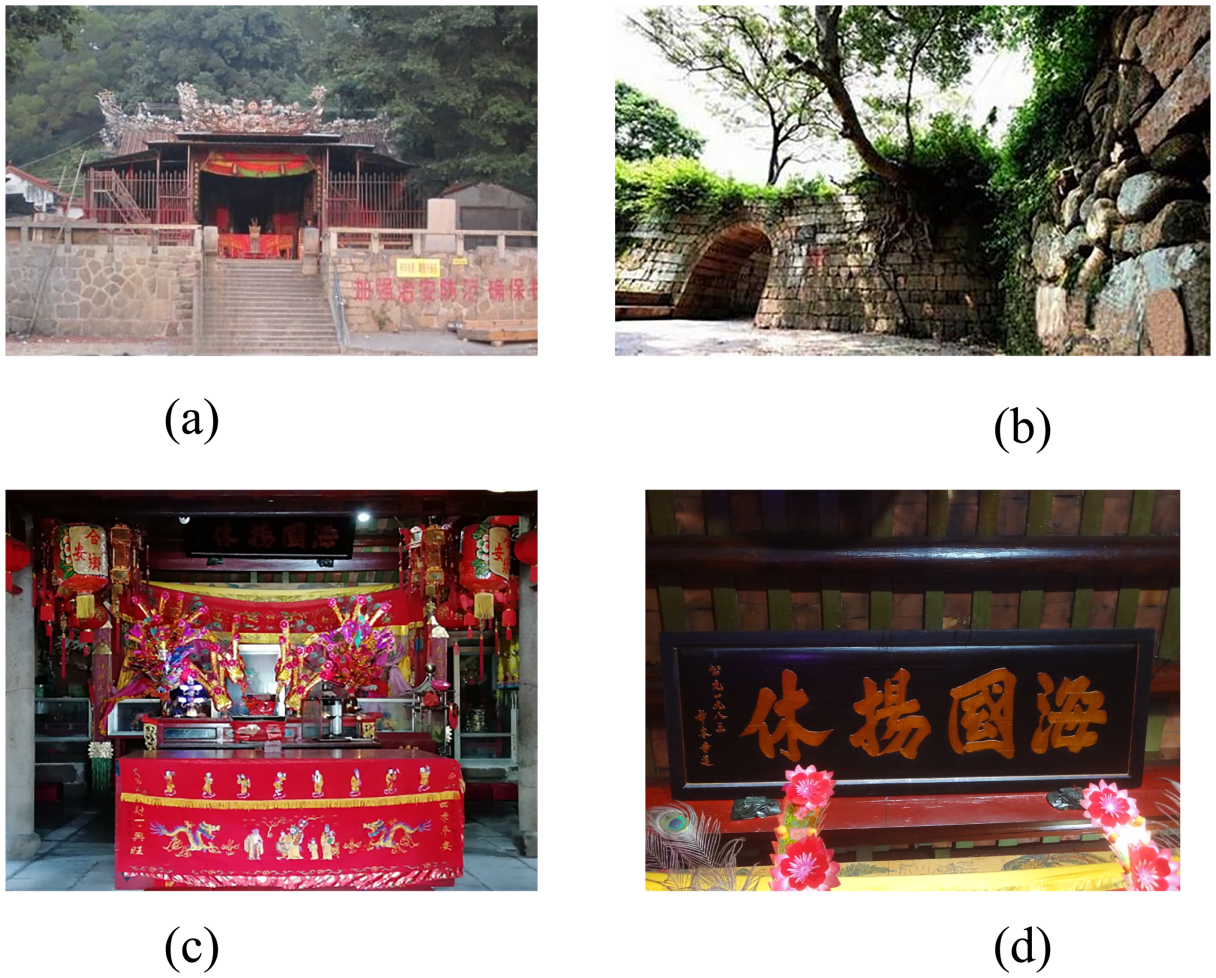
The cultural landscapes in Dongmen village. **(A)** Guandi Temple; **(B)** Xuanzhong Suo town; **(C)** Mazu Temple; **(D)** ‘Hai Guo Yang Xiu’ plaque.

In Dongmen village, cultural heritage construction of the Maritime Silk Road has included human and geographical landscapes such as the Mazu Temple, the Guandi Temple, the cliff carvings of Guolao Mountain, and the ancient military training ground, which are cultural symbols that people have reconstructed, and now serve as a differentiated commodity (Figure 4). *The Beautiful Village Planning of Dongmen Village (2017–2030)* proposal recommends the formation of a functional structure of “one center, one axis, and two areas,” to bring the Xuanzhong Suo town heritage into the entire Dongmen village; the plan includes a protected area, a tourism area, a new village development area, and a comprehensive service center. This means that in the process of cultural heritage construction, Dongmen village has a clear plan, and ‘renovation’ and ‘tourism’ are concepts that are constantly mentioned in the planning. As an example of the sustainability of cultural heritage behaviors, Wu Youjiang, described the role of place attachment feelings as the force of homeland construction.

*Xuanzhong Suo town is my hometown. I’ve always been interested in the history and culture of my hometown. Over the years, I have consulted historical books about Suo town and visited many scholars who understand history and culture. Three years ago, a group of Taiwanese tourists came to visit us. The residents thought I knew more about Xuanzhong Suo town and asked me to explain it. Later, whenever someone visited Xuanzhong Suo town, the village would ask me to show them around, so the village also let me set up a tourist information consultation service department. I was obliged to lead the way for many experts who came to my homeland to investigate, and I also discussed a lot about the history and culture of Xuanzhong Suo town with them. Over the past three years, I have hosted thousands of visitors* (Wu Youjiang, Male, 57 years old).

When residents with strong feelings of place attachment realize that their homeland is expected to enter the discourse on cultural heritage as a core area of the Maritime Silk Road, they are proud of their hometown, because this means that their cultural heritage is being recognized by the outside world. However, we also found some differing opinions. One participant expressed concerns:

*Although the changes in the environment are exciting, especially as the construction of cultural heritage brings development opportunities for some, like developers and planning departments, most of us haven't seen the so-called detailed restoration plans during the renovation. What I want to see is cultural heritage with a sense of history, not something fake. Similarly, some people, because of their fame, have gained most of the hosting opportunities. I think the rest of us should also have the chance to explain the history of our hometown* (Lin Xiaoyu, Female, 38 years old).

Tourism is a concept that is constantly mentioned in planning. Commercial pedestrian streets, tourist service stations, social parking lots, tourist avenues, leisure seats, four-corner pavilions, and other facilities that facilitate tourists are embedded in Dongmen village. In China’s traditional rural societies, these do not exist, and there is no need for facilities such as tourist service stations. These planning concepts directly point to serving heritage tourism. The planning concept of ‘tourism’ shows that Dongmen village is changing from a community of acquaintances to a place that welcomes others. The heritage planners are trying to reorganize and package the cultural resources to cater to consumers’ imaginations of the Xuanzhong Suo town of the Ming dynasty, and to meet consumers’ needs. The reconstruction or adjustment of these external landscapes not only facilitates the development of tourism in Dongmen village on a surface level but also deeply reshapes the emotional connections among participants. According to Korpela et al. (2001), the formation of place attachment is closely tied to an individual’s sense of identification with their environment. By introducing tourism-related facilities into Dongmen village, residents have not only functionally adapted to the new environment but have also emotionally reassessed their relationship with the place. As cultural resources are repackaged, residents have transitioned from passive recipients of culture to active participants, imbuing the place with new meanings and values. This dynamic process demonstrates that tourism planning is not merely the reconfiguration of space but also the reconstruction of emotional and social relationships, ultimately forging stronger community bonds and deeper cultural identity (Basile et al., 2021).

Dongmen village’s reconstruction of its cultural heritage, particularly its historical ties to Xuanzhong Suo town and the Maritime Silk Road, has significantly influenced local residents’ sense of place attachment. This process of re-evaluating and revitalizing cultural elements (ranging from the preservation of old ruins to the introduction of new tourism facilities) serves not only to maintain historical continuity but also to reshape the residents’ emotional connections to their homeland. The reconstruction efforts allow locals to see their environment not merely as a space of daily life, but as a carrier of historical and cultural identity (Yang et al., 2022). This shift reinforces pride in their heritage, deepening their attachment to the village (Qin and Leung, 2021). With the gradual reconstruction of cultural heritage, residents feel that their cultural traditions are respected, which strengthens emotional bonds and encourages their more active participation in heritage preservation (Wu et al., 2021). However, the transition from a traditional living environment to a cultural heritage tourism destination also presents challenges, as some residents feel ambivalent toward changes that prioritize the needs of external tourists while neglecting the authenticity of local culture. This complex interaction highlights how place attachment is both reinforced and reshaped by cultural heritage construction, emphasizing the critical role of emotional and cultural connections in sustaining local heritage.

### 3.2 Historical memory and folk beliefs: strengthening place attachment through heritage narratives

Historical memories are closely linked to marine cultural heritage construction. During the investigation, it was found that in the process of using historical memory, legends, and heritage landscapes closely related to the Ming dynasty were reawakened and reorganized by residents, and continuously spread to the outside world. ‘Suo town of Ming dynasty’, a label with significant local characteristics, contains the precious historical memory of the residents integrating into the Maritime Silk Road, and it is the most imaginative space for consumers, so it has become an attractive place for capital inflows. Michael Herzfel argues heritage is the central theme of the official discourse of historical conservation, heritage evokes notions of a material inheritance given in trust for future generations (Herzfeld, 2019). His concept of heritage allows us to consider the critical issue in the relationships between local inhabitants, responsible behaviors, tourist destination and heritage site building, participatory processes.

The histories and legends of Xuanzhong Suo town during the Ming dynasty were consciously awakened and reorganized, affecting the actions of residents in the marine cultural heritage construction. Today, there are still several sections of the circumvallation and three gates at the former site of Xuanzhong Suo town. The plaque on one gate is inscribed ‘The First Battalion of Southern Fujian’, which expresses the important position of Xuanzhong Suo town in the coastal defense forts system. In recent years, historical relics such as the circumvallation and gates have been restored. According to the concept of ‘restoring the old as the old’, the heritage planners have preserved the historical appearance of the ancient circumvallation and gates, and the construction of the cultural landscape of Xuanzhong Suo town from the Ming dynasty has been in line with heritage construction.

According to historians, military officers with creative initiatives like Qi Jiguang, whose strategy had also been successful in defending the border, were a major factor in the Chinese forces’ victory over the marauding pirates. Evidently, his reputation and legacy continued in China (Torck, 2015). For residents of Dongmen village, the historical memory ‘During the Jiajing period, Qi Jiguang fought against Japanese pirates and bandits, and once stationed troops in Xuanzhong Suo town’ has been reconstructed and presented to the outside world in many forms such as wall paintings and sculptures. During the fieldwork, it was discovered that locals had spray-painted paintings on the walls of modern houses with the theme of Qi Jiguang’s fight against the Japanese pirates, trying to show tourists the history of the area’s fight against the Japanese. When the director of the Mazu Temple spoke about the history of the temple, he deliberately emphasized two plaques hanging on the beams of the main hall, inscribed ‘Tian Shang Sheng Mu’ (天上聖母) and ‘Hai Guo Yang Xiu’ (海國揚休), which were handed down from the Ming dynasty (Figure 4). In their daily lives, the residents have various historical objects such as foreign ancient coins handed down from their ancestors, which support the history of active maritime trade in Xuanzhong Suo town. During the investigation, one participant (Wu Youliang, male, 35 years old) informed us that there are a total of 29 stone inscriptions on Mount Guolao, which include poetry and travelogues attributed to several military leaders from the Ming Dynasty. Among these, three inscriptions belong to the renowned coastal defense general Zhang Yuanxun. His descendants periodically visit this site to perform rubbings, seeking to commemorate the heroic deeds of their ancestors, and they display the inscribed texts within the village.

Among the many cultural heritage sites, the Guan Di Temple has gained prominence due to its large group of believers and the widespread reputation of the deity. The Guan Di Temple is located at the foot of Guolao Mountain, with a backdrop of the mountain and the sea. The temple was built in 1378 with donations from residents mobilized by local officials. It has been repaired many times in history, and the last one was restored in 1980 with donations from local residents and overseas Chinese. As the guardian deity of Dongmen village, Guan Di has important cultural significance and practical value in marine cultural heritage tourism. For the fishermen of Dongmen village, Guan Di is regarded as a god with the power to protect the fishermen. Although Guan Di is not a sea god, it is believed that he protects the fishermen since most of the residents who worship him make their living by fishing. The story of Guan Di’s protection of the residents is often recounted, and one of the classic stories is shared by a participant as follows:

*It is said that during the Qing Dynasty, pirates often used this beach to land quietly and then raid the Xuanzhong Suo town. One night, the pirates were preparing to attack the Xuanzhong Suo town, and the residents didn't realize the imminent danger. As the pirates approached the walls of the town, they suddenly saw that the entire wall was lit up with red lights and covered with weapons. The pirates think that the locals have prepared their defense and run away. Later, the pirates asked around why the city walls will have lights and weapons, the local residents did not have any defense preparations beforehand, the pirates realized that the original is Guan Di manifestation* (Huang Ling, Female, 42 years old).

Such legends show that Guan Di belief is closely related to people’s production and life, reflecting the close integration of marine culture and beliefs. Nowadays, when tourists visit the Guan Di Temple, they will not only learn about these interesting legends and experience the fusion of marine culture and beliefs, but also gain a deeper understanding of the history and lifestyle of Dongmen village.

*I think that not only have the historical landscapes been transformed, but these historical legends have also been somewhat 'enhanced.' For example, as an amateur tour guide, I must learn and train to tell these stories. We cannot present distorted versions of the tales to visitors, but we also need to make them engaging, so sometimes we exaggerate the mythical aspects. Even though it’s for the tourists, I feel like I’m really telling the stories to myself. If I were to explain something in a very dilapidated state, I think it would only evoke a sense of sadness in me. So perhaps reasonable restoration is useful, as it at least makes things visually pleasing. As the saying goes: 'Every place has something that evokes nostalgia; don’t underestimate this sense of happiness, as it can encourage people to stay.' Seeing my hometown become more beautiful also gives me a sense of happiness* (Huang Ling, Female, 42 years old).

Among the marine culture, the Mazu belief plays a significant role. Huang Musheng (male, 43 years old) informed us that during specific festivals, the Mazu Temple attracts numerous visitors. Particularly on Mazu’s birthday, the entire village is immersed in a vibrant atmosphere. On this day, each family brings offerings to pray for blessings, while opera performances become a prominent recreational activity for three consecutive days. Moreover, Taoist rituals are an integral part of the village’s traditions. A three-day ceremony takes place at the onset of winter in the Year of the Rat, Dragon and Monkey, respectively. According to locals, as winter arrives and agricultural work concludes, farmers express gratitude to deities for bountiful harvests and seek peace in the upcoming year. These cultural practices not only reinforce the historical significance of the Maritime Silk Road but also foster residents’ increased awareness and involvement in preserving local intangible cultural heritage. Simultaneously, engaging in festival activities offers visitors a novel cultural experience that connects Dongmen village with its maritime cultural heritage.

Through the exploration of historical memory and folk beliefs, we can observe the crucial role these cultural elements play in enhancing place attachment and promoting cultural sustainability (Hussein et al., 2020). For instance, the reconstruction of the history and legends of Xuanzhong Suo Town by residents has created a shared cultural narrative, which not only strengthens their emotional dependence on the place but also inspires their active participation in the preservation of marine cultural heritage. The reconstruction of historical landscapes and the revival of traditional beliefs, such as the worship of Guan Di and Mazu, enable residents to maintain material heritage while also transmitting intangible culture, thereby forming emotional ties across generations.

Historical memory enhances residents’ emotional attachment to the place by reconstructing and disseminating stories related to local culture (Gao et al., 2020). This emotional reliance motivates residents to actively engage in the protection and transmission of cultural heritage, fostering a sense of responsibility (Cai and Cheng, 2024). At the same time, folk beliefs, such as the reverence for Guan Di, not only reflect cultural identity but also play a cohesive role in social life. Through participation in festival activities and religious rituals, residents reinforce their sense of belonging to local culture, and this engagement further encourages them to adopt sustainable actions in their daily lives, such as participating in local environmental protection and cultural heritage maintenance. In these ways, historical memory and folk beliefs not only strengthen place attachment but also lay the foundation for cultural sustainability, demonstrating their significance in contemporary society.

### 3.3 Place attachment as a driving force for sustainable homeland development

In this series of sustainable behaviors, feelings of place attachment by the residents of Dongmen village to their homeland play an important role. Human geographer Tim Cresswell sees that home is an exemplary kind of place where people feel a sense of attachment and rootedness. Home, more than anywhere else, is seen as a center of meaning and field of care (Cresswell, 2004).

The residents who were born and raised in Dongmen village have a deep attachment to their homeland, and these emotions have profoundly influenced its cultural landscape. This is evident in the efforts of the local community to rebuild the Mazu Temple. Although the temple has suffered multiple instances of damage, it has been preserved through ongoing reconstruction or restoration driven by the residents’ sense of place attachment. According to the 2012 Mazu Temple Reconstruction Tablet and the draft of Dongmen village history compiled in the township archives, after the construction of Xuanzhong Suo town in 1387, the local people built seven Mazu Temples to seek the deity’s blessings. However, six of these temples fell into ruin due to invasions by Japanese pirates and robbers. To protect the Mazu statues, they were relocated to the present-day temple for worship. The structure was subsequently rebuilt twice during the Qianlong and Daoguang periods of the Qing dynasty.

In the 1970s, the Mazu Temple was destroyed again. With full enthusiasm for cultural heritage and protecting ancient buildings, Chen Huaguo, a sage from Dongmen village, volunteered to be responsible for reconstructing the Mazu Temple. He led the residents to rebuild it in 1982, taking into consideration the memories of the Temple by several elderly people. According to historical records, seven statues of Mazu were reshaped and placed in the temple. According to Chen Huaguo’s statement:

*In the process of being in charge of rebuilding, I needed to coordinate various relationships, go through various procedures, and suffered a lot. If I didn’t have the sense of mission to do something practical for my hometown and history, it would be difficult for me to stick to it. Fortunately, I have served as a cadre in the village, so I could communicate and coordinate with relevant units more conveniently, so that the reconstruction of the Mazu Temple did not encounter too much resistance* (Chen Huaguo, male, 50 years old).

Regarding Chen Huaguo’s behavior, his mother also supported him. She encouraged him to rebuild the Mazu Temple as a good deed to local cultural heritage, which should be done without hesitation, regardless of other people’s views and difficulties. During the rebuilding process, many overseas Chinese living in Nanyang offered to provide financial or networking support, and therefore, a new Mazu Temple was rebuilt after only a few months. The local elders were grateful to Chen Huaguo when they witnessed the memory of the Mazu Temple reappearing in their hometown. To a certain extent, it is because of their feelings of place attachment to the cultural landscape of their homeland that the residents actively participated in the practice of cultural heritage.

In a marine community, residents have frequent interactions with the ocean, and the convenient sea transportation prompts residents to migrate overseas. Immigrants or diaspora individuals deepen their emotional connection to their homeland through rediscovering the traditional culture of their native land or relying on family ties (Du, 2017; Zou et al., 2021). For discrete groups of individuals, the cultural heritage that represents their past can evoke memories and sense of identity with their hometown; cultural heritage is important and serves as a stable link between their intergenerational inheritance. According to William Safran, such individuals retain a collective memory, vision, or myth about their original homeland’s physical location, history, and achievements; they continue to relate, personally or vicariously, to their homeland in one way or another, and their ethno-communal consciousness and solidarity are importantly defined by the existence of such a relationship (Safran, 1991).

Dongmen village belongs to Meiling town, and Meiling has more than 10,000 overseas Chinese, Hong Kong, Macao, and Taiwan compatriots. The superior geographical conditions of the peninsula have facilitated the migration of masses to overseas. Therefore, before the founding of the People’s Republic of China, many people lived in Nanyang, making its population the largest in the county. Meiling people have a wide distribution of overseas Chinese, including Singapore, Malaysia, Thailand, America, Canada, Cambodia, Vietnam, and other countries. Although people living overseas have been separated from their ancestral homelands for decades, people still have a place attachment to their ancestral homelands (Huang et al., 2018). This emotion has prompted them to care about the construction of their ancestral home, for example, activities such as co-repairing ancestral temples and repairing ancestral houses are the most direct manifestations of their feelings of place attachment.

*Due to our increased communication in recent years, my overseas relatives’ feelings for their homeland seem to have deepened through our exchanges as we reminisce about shared memories. When they see Dongmen village through video, they experience a sense of nostalgia—a desire to return, yet unable to do so. Consequently, many support the restoration of the ancestral hall by transferring money via WeChat and request that the agents provide updates on the restoration progress and how the funds were utilized, seeking confirmation once the work is completed. This process also seems to validate their own sense of longing for home* (Liu Yunfei, male, 65 years old).

Many well-preserved buildings from the Ming and Qing dynasties in Dongmen village are inseparable from the sustainable behaviors of residents and overseas Chinese. For example, the Liu clan has nearly 100 members in Dongmen village, although the Liu clan has moved to Southeast Asian countries such as Malaysia, the ancestral hall is an important link to maintaining the clan. During the 2015 renovation of the Liu clan’s ancestral home, some of the funds came from Malaysian clansmen, amounting to 15,000 Yuan, and the other funds were contributed by each household of Liu’s family in Dongmen village, i.e., 1,000 Yuan. After the repair was completed, more than 10 people from Malaysia came to worship their ancestors. This example demonstrates that the newly renovated ancestral house unites the Liu clan, enabling the continuous renewal of the heritage landscape of Dongmen village.

In conclusion, place attachment plays a crucial role in driving the sustainable behaviors of residents in Dongmen village. This emotional connection reflects the intrinsic relationship between place attachment and sustainable behavior through multiple mechanisms. Firstly, place attachment significantly enhances residents’ sense of identity with their cultural heritage, making them more inclined to engage in cultural protection and maintenance activities when facing external challenges. For instance, the reconstruction of the Mazu Temple not only serves as a restoration of a historical site but also represents a reaffirmation of local cultural identity. By actively participating in the reconstruction process, residents deepen their understanding of local culture and, in turn, develop a sense of responsibility toward their place.

Secondly, place attachment fosters collective action among residents (Martin, 2003). In the case of Chen Hague, his personal enthusiasm not only motivated his individual efforts but also effectively mobilized surrounding community members to participate in the reconstruction. This phenomenon of collective participation strengthens social ties among residents, creating a shared sense of responsibility for the protection of cultural heritage. It indicates that when individuals recognize the connection between their actions and a common goal, their willingness to participate and motivation for action significantly increase, thus facilitating the achievement of sustainability objectives.

Furthermore, place attachment also plays an important role among overseas Chinese (Li and Chan, 2018). Despite being abroad, their emotional connection to their homeland remains profound, driving them to engage in the protection of cultural heritage through economic support and emotional ties. This phenomenon further illustrates that place attachment is not only a geographical belonging but also a cross-cultural emotional bond that stimulates transnational communities’ concern and involvement in the development of their homeland (Zhu and Airey, 2021), thereby promoting the continuity of sustainable behaviors.

## 4 Discussion

This research shows how a village with a historical depth is understood, interpreted, and used in traditional cultural heritages, and adapted to changes in the concept of sustainability. The findings of this research contribute to a better understanding of the relationship between sustainability cultural heritage behaviors and the cultural heritage construction based on the theory of place attachment and from the perspective of local residents. In Dongmen village, the residents’ historical memories, local knowledge, and feelings of place attachment are continuously reinforcing the history of Xuanzhong Suo town through the reorganization of the environmental and human landscape, thus, linking the residents’ spatial and temporal locations to the core area of the Maritime Silk Road. Through common cultural symbols, historical memories, and collective expressions, the village’s ties to maritime cultural heritage are reinforced. In the process of constructing the maritime cultural heritage of Xuanzhong Suo town, the residents have a new understood their own spatial and temporal locations; they have a new understanding of who they are, where they are, and what responsibilities they should bear, which means that their feelings of place attachment have been re-articulated and deepened.

The findings, which are in line with numerous studies of other species (Lee, 2011; Hoang et al., 2020), point to the fact that people’s attachment to particular places offers numerous opportunities to study sustainable behaviors toward cultural heritage construction (Esperanza, 2024). The findings suggest that place attachment could be useful in explaining sustainable behaviors and pro-environmental behaviors. It appears that such behaviors are more likely to occur when an individual has a positive attachment to a place (Burley et al., 2007; Walker and Ryan, 2008; Csurgó and Smith, 2022; Jiao et al., 2023). Places are fusions of human and natural order and are the significant centers of our immediate experiences of the world. Places are directly experienced phenomena of the lived-world and hence, are filled with meanings, real objects, and ongoing activities. They also carry a certain degree of spiritual significance (Christou et al., 2019).They are often profound centers of human existence to which people have deep emotional and psychological ties (Relph, 1976). This research has shown that places such as Dongmen village are brimming with ongoing activities that are important to local residents. These villages serve as significant points of communal and individual attachment and are frequently profound centers of human existence to which individuals have strong psychological and emotional bonds. In a sense, the residents of Dongmen village understand who they are, where they are, and what responsibilities they should bear in the process of constructing the marine cultural heritage of Xuanzhong Suo town.

In the study of sustainable cultural behaviors among residents of heritage sites, numerous scholars have offered valuable perspectives. For instance, some researchers employ the concept of “heritage responsibility behavior” to elucidate local residents’ identification with their cultural heritage (Zhang, 2014; Ju and Cheng, 2023; Cai and Cheng, 2024; Yi et al., 2024). Specifically, Ju and Cheng (2023) identified five modes that can drive residents to develop high levels of heritage responsibility behavior, which include behavioral attitudes, place attachment, political embeddedness, and cultural embeddedness. The research by Yi et al. (2024) found that local residents’ perception of authenticity has a positive impact on their sense of place identity and cultural self-enhancement, which in turn stimulates their sense of heritage responsibility and increases the likelihood of sustainable conservation behaviors. This study also supports this perspective, suggesting that state-led heritage initiatives, to some extent, enhance the authenticity of these heritages, thereby fostering local residents’ attachment to their hometown. Tan et al. (2018) emphasized that research on place attachment highlights individual feelings or experiences. Scholars have found that the ways in which people develop attachments to historical places can vary significantly. Wang (2023) examined five dimensions of local residents’ attachment to historical areas, including autobiographical, nostalgic, restorative, aesthetic, and intellectual aspects. Fang et al. (2021) found that residents’ participation in environmental protection activities enhances their emotional connection to heritage and subsequently fosters a sense of responsibility, thereby promoting the adoption of heritage conservation behaviors.

Although a growing body of tourism research has investigated residents’ cognitive and emotional connections to heritage destinations, this line of research is largely overlooked in the Dongmen village context. In this study, we found that the sustainable behaviors of Dongmen village residents toward cultural heritage construction were influenced by feelings of place attachment, which subsequently influenced homeland construction actions that, in turn, shaped human-place relationships. For example, the repairs or reconstruction of palaces, ancestral halls, and ancestral houses by the residents show that they have made use of local knowledge and indigenous resources. By proactively using associations of the elderly, faith groups, family networks, and overseas relationships, the residents can participate in cultural heritage practices and can maintain their cultural subjectivity, and thus, protect their cultural heritage.

One of the more significant findings to emerge from this study is that focusing on place attachment should help to consider the critical issue in the relationships between residents, sustainable behaviors, tourist destination and heritage site building and maintenance, participatory processes. In the process of marine cultural heritage construction, the local government and scholars have actively interacted. Scholars have actively proposed the strategy of protecting and developing heritage, and the government has adopted most of the feasible suggestions. The local tourism industry has been fully developed, and residents have benefited from the tourism industry and improved their quality of life, with a per capita income of 12,536 yuan, an increase of 9.8%. For effective sustainability of local residents’ cultural heritage behaviors, heritage planners should focus on the construction of external meanings and the unique spirit of place (Christou, 2021), as well as aim to maintain residents’ feelings of place attachment, their sense of conserving their cultural heritage, and their emotional bond with their homeland.

The results indicate that developing heritage tourism can give residents a sense of self-identity and an emotional connection to their homeland. This is mainly because heritage tourism, when properly managed, can bring awareness of the place’s history, culture, and uniqueness, which may encourage residents to preserve cultural heritage sustainably. The findings of this study imply that, for effective sustainability of residents’ cultural heritage behaviors, heritage planners should focus on residents’ environmentally responsible behaviors, as well as aim to maintain residents’ feelings of place attachment, their sense of conserving their cultural heritage, and their emotional bond with their homeland. Heritage planners need to ensure that the development of heritage tourism supports residents’ place attachment so that it is mutually reinforcing to ensure the long-term sustainability of the destination’s tourism. Furthermore, by being aware of the impact heritage tourism has on the local community, residents can leverage cultural heritage to boost the sustainability of cultural heritage behavior and strengthen place attachment and community identity.

The main findings of this study reveal the critical role of place attachment in cultural heritage protection and sustainable development; however, several limitations remain. First, while qualitative methods can deeply capture participants’ emotions and experiences, the limitation of the sample—where over 80% of participants are aged 30 and above—may affect the generalizability of the results. Therefore, future research should consider incorporating quantitative analysis methods to enhance the verifiability and universality of the findings and expand the diversity of the sample to reflect the phenomenon of place attachment across different groups more comprehensively. Second, although this study explores the influence of historical memory and folk beliefs on place attachment, it has not delved into the heterogeneity of place attachment experiences among various stakeholders. Future research could investigate the role of government in cultural heritage protection, the balance developers strike between cultural preservation and commercial development, the impact of tourists on local cultural identity, and longitudinal studies on how place attachment changes over time. These aspects will contribute to a more comprehensive understanding of the multifaceted influence of place attachment on cultural heritage construction and provide targeted recommendations for relevant policies. Additionally, future studies could focus on group differences, such as those based on generation, gender, or socioeconomic background, to explore their impact on place attachment emotions, thereby enriching the understanding of place attachment.

## Data Availability

The original contributions presented in the study are included in the article/supplementary material, further inquiries can be directed to the corresponding author.
